# Students’ Acceptance of the COVID-19 Impact on Shifting Higher Education to Distance Learning in Poland

**DOI:** 10.3390/ijerph17186468

**Published:** 2020-09-05

**Authors:** Mariia Rizun, Artur Strzelecki

**Affiliations:** Department of Informatics, University of Economics in Katowice, 40-287 Katowice, Poland; mariia.rizun@ue.katowice.pl

**Keywords:** e-learning, distance learning, higher education, online communication tools, legal ordinance, COVID-19, coronavirus, pandemic

## Abstract

This paper is dedicated to the higher education institutions shifting towards distance learning processes due to the global pandemic situation caused by COVID-19 in 2020. The paper covers the pandemic situation in Poland generally, analyzing governmental ordinances and tracking the gradual extension of restrictions for educational institutions. The purpose of this study is to investigate the influence of Experience, Enjoyment, Computer Anxiety, and Self-Efficacy on students’ acceptance of shifting education to distance learning. The study tested and used the adapted General Extended Technology Acceptance Model for E-Learning (GETAMEL) in the context of coronavirus pandemic. The partial least squares method of structural equation modeling was employed to test the proposed research model. The study utilizes an online survey to obtain data from 1692 Polish undergraduate and graduate students in both full- and part-time study. The dataset was analyzed using SmartPLS 3 software. Results showed that the best predictor of student’s acceptance of shifting education to distance learning is Enjoyment, followed by Self-Efficacy. Both Perceived Ease of Use and Perceived Usefulness predict student’s Attitude Towards Using and Intention to Use the distance learning. The findings improve understanding regarding the acceptance of distance learning and this work is therefore of particular interest to teachers and practitioners of education.

## 1. Introduction

On 31 December 2019, the World Health Organization (WHO) China Country Office was informed of cases of pneumonia of unknown cause detected in Wuhan City, Hubei Province of China [1]. On 20 January 2020, 282 confirmed cases of 2019-nCoV were reported already from four countries: China, Thailand, Japan, and the Republic of Korea. [2]. Finally, the last (as of 13 July 2020) WHO report informs of 12.91 million cases of COVID-19 disease globally, with 561 thousand deaths [3]. Additionally, in March 2020, WHO announced COVID-19 outbreak of a global pandemic [4]. Since that moment, cities and transportation have been brought into shutdown, with many business and educational activities as well as people’s everyday lives being temporarily “suspended” [5].

### 1.1. Global Education Policies

The “pandemic vs. education” situation has already been widely analyzed by researchers all over the world. For instance, Raaper and Brown state that the shift towards online education has a significant impact on students’ mental and physical wellbeing, making students’ social networks an essential tool of support during the pandemic period [6]. Additionally, the authors have calculated that universities in the United Kingdom may experience a finance loss of up to £2.5 billion. Continuing on the topic of funds, Ahlburg has suggested that COVID-19 affects, first of all, universities with tenuous financial situation—for instance, those highly dependent on external funding or on enrollment of international students [7].

Nash and Churchill raise the interesting subject of academic women’s occupation during the pandemic [8]. It is observed that women’s publication track records have decreased significantly, even by 50% in some areas. It is explained by the fact that, except for daily routine women have at home, they now have to prepare and record online lectures—sometimes only having the option to do it at night when their children are asleep. In a survey of 441 university students exploring the COVID-19 impact on learning, Olmos-Gómez has found out that (among others) 75.25% of the respondents were able to solve a problem that arises with the help of tools like email or Facebook and 78.75% consider that their ties with family and friends have become stronger during self-isolation [9]. Studying the impact of social media in universities worldwide, Obaid AI-Youbi et al. have analyzed the Twitter account of a university in Saudi Arabia and revealed that, in the pandemic period, this account was successfully used as a powerful tool for communicating critical policy-making as well as issues related to teaching and learning [10]. Twitter messages about staying safe at home and continuing to be productive during the pandemic helped to maintain positive attitudes among the students; by providing flexible communication and positive messages to students, the university has built a stronger feeling of belonging to an institution.

In their study, conducted in China in February 2020, Wang and Zhao addressed the anxiety of university students, connected with online learning [11]. From a survey with 3800 respondents, they revealed that university students have a much higher level of anxiety than the general population after the virus outbreak, with a higher percentage of medical students (compared to the general university students) and with female students showing more anxiety than men. Zhang et al. have also analyzed the Chinese education situation, referring to the policy of “Suspending Classes Without Stopping Learning”, which included 5 major moves: provision of network service resources, training teachers, enabling all schools to carry out classes online, formulating guidelines for transition back to traditional education, and developing a plan for schools reopening after the pandemic [12].

On the other hand, Watermeyer et al. have conducted a survey on academic teachers, studying how they react on the move to online teaching [13]. They have found out, for instance, that 60.6% feel confident or strongly confident in their ability to facilitate online teaching and assessment, with those from computer sciences and education being more confident; 72.7% consider their institutions to be supportive in facilitating the move to online learning and teaching; 81.7% state they can access appropriate technologies to support their online teaching and assessment. Researchers Nuere and de Miguel, after observing two universities in Spain during the pandemic, have come to the following conclusions (among others): universities that were used to conducting online classes before have minimal problems working in new conditions; teaching/learning online is particularly problematic for courses like drawing, chemistry, or electronics (were presence in the laboratory is required); the quality of online teaching tools strongly affects the quality of the process [14].

Ebner et al. suggest assessing the readiness of a university for e-learning by using the “seven S model”, i.e., by analyzing strategy, structure, systems, style/culture, staff, skills, and shared value of a university [15]. Finally, the paper explains how a university in Austria has shifted its education process to an internal online platform. Additionally, the authors mention that in Germany, a large group of university professors states that they could not teach online. Thatcher et al., analyzing the situation in Australian universities during COVID-19, claim that COVID-19 negatively affects the number of international student enrolments at universities not only in Australia but all over the world; a decrease in these enrolments at Australian universities below their 10-year average will cause a decrease in total country’s university revenue; COVID-19 might have a negative effect on the level of employment at universities [16]. Tiejun describes the situation in Chinese colleges and universities after the coronavirus outbreak [17]. The plan of “Internet + Education”, which was developed in advance, was deployed at education institutions, which included organization of online learning process as well as plans for comfortable return (when possible) to traditional form of learning considering the epidemic prevention procedure. It is also mentioned that one of the online education platforms in China has gathered 400 million users by 11 February 2020; usage of other widely used platforms has also increased significantly. Generally, it is stressed that due to prior preparation, Chinese education has not suffered from the pandemic and all classes were taught with high quality.

### 1.2. Polish Education Policy

This article is dedicated to the situation in higher education in Poland where, as of 30 April 2020, the notification rate for COVID-19 was 33.2 per 100,000 inhabitants [18]. As well for any other country, in Poland, the education segment is being rather strongly influenced by the pandemic [19]. The global health crisis has forced higher education institutions (HEIs) to replace face-to-face education by distance education [20]. It is obvious that some HEIs were more prepared to such an immediate shift while others, on the contrary, had to react quickly and turn their didactic process almost upside-down to meet the realities of online education [21].

Discussion on how HEIs in Poland are managing this situation will be conducted on the example of the University of Economics in Katowice (UEK), the province of Silesia, southern Poland. Since April 2020, Silesia is the region with the most confirmed cases [22]. UEK is the biggest and oldest business school in the region, one of the top universities in Poland. Each year, over 10,000 Polish and international students follow degree programs at the Bachelor, Master, Doctoral, and Post-Diploma levels at five faculties at UEK: Business, Finance and Administration, Economics, Finance, Informatics and Communication, and Management [23].

The first ordinance on suspension of education activities because of COVID-19 was issued by the Polish Ministry of Science and Higher Education on 11 March 2020 [24]. Following the ordinance, the functioning of HEIs was limited for the period from 12 March until 25 March 2020: for full- and part-time first-and second-cycle studies, as well as for postgraduate and doctoral studies [25]. It was stressed that learning could be implemented using distance learning methods and techniques regardless of whether it was provided in the curriculum, with preservation of the same ECTS (European Credit Transfer and Accumulation System) credits for all courses [26]. The same day the Rector of UEK issued the Ordinance [27], which suspended the learning process at UEK until 25 March and implemented obligatory application of IT tools for the online education process. Moreover, on 11 March, the Rector set up the Crisis Management Committee [28] to coordinate activities on prevention of the spread of COVID-19 disease. Additionally, on 12 March, the Ordinance [27] was issued, prohibiting gatherings of more than 10 people on the University’s campus.

On 20 March, with the Rector’s Ordinance [27], suspension of the learning process was prolonged until 10 April 2020. It was stressed that Bachelor and Master diploma exams and defense processes were not to be conducted until April 10. This was preceded by the ordinance of the Ministry, which amended the previous ordinance, and suspended the traditional learning process until 10 April 2020 [24].

Finally, on 9 April 2020, the Minister of Education of Poland issued an ordinance amending the two previous ordinances and suspending the learning process at educational institutions until 26 April 2020 [29]. This was followed by the Ordinance [27] of the Rector of UEK, which suspended all traditional forms of education (i.e., happening in University’s campus) until 8 June 2020, which is the last day of summer semester 2019/2020 at UEK. A few days later, on 17 April 2020, the Rector issued the Ordinance [27], commanding Bachelor and Master diploma exams and defense processes to be conducted through online communication.

Summarizing, it can be stated that online education at the University of Economics in Katowice (as well at all Polish education institutions) replaced traditional education in the middle of March 2020 and, as of 11 May 2020, will be holding its position until almost mid-June— totally within 89 days.

The first reaction of the authorities of the University of Economics in Katowice to the necessity of shifting towards distance education was noted on 13 March 2020. The University staff was informed (by email) about the works initiated to facilitate the process of distance education and implement friendly online tools for academic teachers and students. The strategy, adopted by the University, had to include four stages of transition to the distance online learning process [28]. At the first stage (around 13 March 2020), all students and teachers gained access to platforms that enabled the realization of classes remotely. The two platforms offered for the distance learning process at UEK were Moodle (Moodle Community, West Perth, Australia) and Google Classroom (Google, Mountain View, CA, USA). It is necessary to stress that both platforms have been applied at the University before but on a much lower scale, since they were not obligatory. To facilitate communication of teachers with students, email addresses were assigned to all student groups, enabling faster direct contact with students within each particular course.

At the second stage (around 30 March 2020), the emphasis was placed on the necessity of live communication of teachers and students—using Google Meet (Google, Mountain View, CA, USA), Google Hangout (Google, Mountain View, CA, USA), Moodle, Microsoft Teams (Microsoft Corporation, Redmond, WA, USA), Skype (Skype Communications S.a.r.i., Palo Alto, CA, USA), and others. On the UEK website [28], a webpage was created containing detailed instructions (text and video) on the usage of G-suite tools (Gmail (Google, Mountain View, CA, USA), Google Meet, Google Forms (Google, Mountain View, CA, USA), Google Drive (Google, Mountain View, CA, USA), Google Classroom, etc.).

Within the framework of the second stage in the middle of April 2020, a survey was conducted among UEK teachers with the objective of revealing what IT communication tools were preferred by teachers within the distance learning process. The authors assume that the UEK authorities have done this research for two major reasons: (1) to find out which IT tools to focus on in the current semester and to provide up-to-date technical support for teachers, facilitating their work in all possible ways; (2) to set a precise plan for distance education in the following semesters—in case the pandemic situation forces HEIs to continue distance education. These (and other possible) objectives would help the University provide its teachers and students with the highest possible comfort of conducting/taking classes online and, as a result, maintain the high level of education regardless of the external conditions.

The third stage (around 2 April 2020) was dedicated to the preparation of detailed rules for conducting semester exams using online solutions—in case this will be required due to the pandemic situation. It was stated that application of the abovementioned IT communication tools allows students to obtain the necessary course credits within the framework of distance learning [28].

Stage four (around 22 April 2020), which is final as of 11 May 2020, as a continuation of stage three, planned realization of diploma exams with online communication tools. On 5 May 2020, the first four diploma defenses were conducted at UEK via Google Meet [30]. Information on the realization of the stages described above can be found on the webpage “UEK during coronavirus” on the University website. As of 11 May 2020, the page is only available in Polish language. The page contains links to the major blocks of information connected with distance learning at the University, like Rector’s Ordinances, a guide for students and academic teachers, exams, diploma exams, video instructions on IT tools, library, research activity, FAQ, etc.

The objective of this research is to analyze the attitude of the UEK students towards the policy of transition to distance learning, in particular, to find out whether they accept (and to what extent) the IT communication tools applied by the University for distance learning. To realize this objective, the authors have conducted a survey among University students.

The paper is organized as follows. In Section 2, the methodology of analysis (partial least squares structural equation modeling (PLS-SEM) and Technology Acceptance Model (TAM)) is presented and hypotheses are drawn. In Section 3, results of the survey as well as PLS-SEM analysis are given. In Section 4, the authors discuss the results of the research and its practical implications, contributions, and limitations. Finally, in Section 5, the overall conclusions drawn from the work are presented.

## 2. Materials and Methods

Structural equation modeling (SEM) is, first of all, a set of techniques for exploring relationships between variables. A version of this method, partial least squares (PLS) regression, enables testing for a small sample and leads to the prediction of indicators. It also allows putting forward hypotheses for the variables with a complex impact on particular aspects of the model. It is important to mention that the variables can be labeled as factors of measured variables. The discretion of modeling enables a new view of the existing theories.

### 2.1. Hypotheses Development

#### 2.1.1. Perceived Usefulness

The Perceived Usefulness (PU) of technologies (in this case, tools applied for university distance learning during the pandemic) is one of the most important elements in the Technology Acceptance Model (TAM). The PU is understood as the degree to which a user of a particular system believes that it would improve his/her work or study performance as compared to alternative methods of carrying out this user’s tasks [31,32]. Perceived Usefulness influenced the decision of a user on whether to accept or reject the particular technology. In accordance with the original TAM [33], user’s PU influences his/her Attitude Towards Using and Intention to Use technology. In the context of distance learning, to which universities have had to switch because of the pandemic, the authors’ have used the TAM principle to formulate the following hypotheses about the PU.

**Hypothesis** **1** **(H1):** *Perceived Usefulness of distance learning positively affects students’ Attitude Towards Using distance learning tools.*

**Hypothesis** **2** **(H2):** *Perceived Usefulness of distance learning positively affects students’ Intention to Use tools for distance learning.*

#### 2.1.2. Perceived Ease of Use

The Perceived Ease of Use (PEOU) of technology is considered as the degree to which a person, using any system, believes that this usage would be effortless [34]. According to the TAM concept, Perceived Ease of Use of technology is one of the predictors of user’s Attitude Towards Using the technology, subsequent behavioral intentions, and actual usage [35]. When users’ perceptions of the ease of use and the usefulness of the technology are positive, they will embrace new technology without any problems [36]. According to [33], the PEOU (as well as the PU) affects user’s Attitude Towards Using and Intention to Use technology. However, some researches that used TAM to analyze e-learning technologies have given contradictory results. In one case, the PEU was not a good predictor of the Intention to Use a learning management system; in the other case, it had a significant influence both on students’ Attitude Towards Using and on Intention to Use [31]. Despite the possible contradictions, the authors have set two hypotheses for the students’ PEOU of distance learning tools.

**Hypothesis** **3** **(H3):** *Perceived Ease of Use of distance learning tools positively affects students’ Perceived Usefulness of distance learning.*

**Hypothesis** **4** **(H4):** *Perceived Ease of Use of distance learning tools positively affects students’ Attitude Towards Using distance learning tools.*

#### 2.1.3. Attitude Towards Using

The survey conducted in [35] showed that Attitude Towards Using (ATU) had not affected the Intention to Use (among 122 college students). This is explained by the difference in technologies and user population. It is also suggested that a positive perception of the usefulness of technology is more important than the attitude towards applying this technology. Nevertheless, following the TAM principle (the ATU influences the ITU), the hypothesis about ATU was put forward in this research.

**Hypothesis** **5** **(H5):** *Students’ Attitude Towards Using distance learning tools positively affects their Intention to Use tools distance learning tools.*

#### 2.1.4. Intention to Use

It is considered that the user’s Intention to Use (ITU) technology affects his/her decision on whether to actually use it (Actual Use). TAM adaptation for the examination of students’ satisfaction and technology adoption in online classes allowed for finding out that students’ Intention to Use technology influences their learning outcomes in the online class environment [37]. All these facts allowed the authors to formulate the next hypothesis.

**Hypothesis** **6** **(H6):** *Students’ Intention to Use tools distance learning tools positively affects the Actual Use of distance learning tools by students.*

#### 2.1.5. Actual Use

The Actual Use (AU), i.e., the act of applying something [34], is the final element of the TAM, which states the fact of a user making (or not) use of any technology. Being the final step in the chain of technology acceptance, the AU does not have any influence on other constructs of this model.

#### 2.1.6. Experience

In the year 2016, Abdullah and Ward [38] extended the well-known version of the TAM model, developing a General Extended Technology Acceptance Model for E-Learning (GETAMEL). The authors of GETAMEL elaborated on the group of TAM external factors, complementing it with five constructs: Experience, Subjective Norms, Enjoyment, Computer Anxiety, and Self-Efficacy. In the work, computer Experience (XP) is defined as the amount and type of computer skills acquired by a person over time. XP is one of the most important external factors. Individuals with higher computer-related skills are more likely to have more positive feelings about the usage of any online/distance learning tool [36,38]. The above-stated has allowed the authors to put forward two hypotheses.

**Hypothesis** **7** **(H7):** *Experience of students in the process of distance learning has a positive effect on their Perceived Usefulness of distance learning.*

**Hypothesis** **8** **(H8):** *Experience of students in the process of distance learning has a positive effect on their Perceived Ease of Use of distance learning tools.*

#### 2.1.7. Enjoyment

Following the GETAMEL [38], Enjoyment (ENJ) is understood as the extent to which the activity of applying any system is perceived to be enjoyable, regardless of the consequences of this system’s usage. It was also revealed in [32] that a system that is found enjoyable is more likely to be perceived as easy to use and useful, and user’s intention to apply it gets a higher degree. Many studies have demonstrated that Enjoyment from using any system affected users’ PEOU. Researchers also observe a strong positive relation between the Enjoyment and Perceived Usefulness of online learning systems, which increases students’ intention to use (Actual Use) of these systems [39,40]. Referring to these conclusions, the authors propose two research hypotheses as for the Enjoyment of distance learning tool during the pandemic.

**Hypothesis** **9** **(H9):** *Students’ Enjoyment of the process of distance learning has a positive effect on their Perceived Usefulness of this process.*

**Hypothesis** **10** **(H10):** *Students’ Enjoyment of the process of distance learning has a positive effect on their Perceived Ease of Use of distance learning tools.*

#### 2.1.8. Computer Anxiety

According to [41], Computer Anxiety (CA) is characterized as the invoking of anxious or emotional reactions when it comes to performing any activity on computer. For Anxiety, as the only construct with negative sentiment, the hypotheses were put forward to prove or disprove the CA negative influence on students’ perception of technology [42]. It is also necessary to mention that some research, like [39], has shown that CA does not have a significant relationship with Perceived Usefulness and Perceived Ease of Use.

**Hypothesis** **11** **(H11):** *Students’ Computer Anxiety, felt in the process of distance learning, has a negative effect on their Perceived Usefulness of distance learning.*

**Hypothesis** **12** **(H12):** *Computer Anxiety, felt in the process of distance learning, has a negative effect on their Perceived Ease of Use of distance learning tools.*

#### 2.1.9. Self-Efficacy

Computer Self-Efficacy (SE) is defined as a user’s belief about his/her ability to conduct a particular task using a computer. Findings also suggest that students who have higher e-learning SE are more likely to use e-learning and computer-supported education [39,43]. SE is considered to have high influence especially on the Perceived Ease of Use [40]. Therefore, the authors propose the following hypotheses.

**Hypothesis** **13** **(H13):** *Students’ feeling of Self-Efficacy, experienced while learning distantly, positively affects their Perceived Usefulness of the process.*

**Hypothesis** **14** **(H14):** *Students’ feeling of Self-Efficacy, experienced while learning distantly, positively affects their Perceived Ease of Use of distance learning tools.*

### 2.2. Methodology

The process of evaluation of results of the partial least squares structural equation modeling (PLS-SEM) involves two steps [44]. In step 1, the examination of reflective and formative measurement models is conducted. This is a necessary part of the evaluation because it provides support for the measurement quality. When quality is confirmed, the structural model evaluation is conducted in step 2 [45]. While in step 1, the measurement theory is examined, step 2 covers the structural theory that involves testing the proposed hypotheses and that addresses the relationships among the latent variables. Our model contains only reflective measures. The “Actual Use” construct is included neither in the reflective nor in the formative measurement model assessment. It is a single-item construct. For this construct, the indicator data and latent variable scores are identical. Consequently, the AU does not have a measurement model, which can be assessed using the standard evaluation criteria.

At this stage, we start by examining the indicator loadings. Loadings above 0.70 indicate that the construct explains more than 50% of the indicator’s variance, demonstrating that the indicator exhibits a satisfactory degree of reliability. The constructs’ internal consistency reliability was assessed. For the composite reliability criterion, higher values indicate higher levels of reliability. Results between 0.70 and 0.95 represent “satisfactory to good” reliability levels [45]. Cronbach’s alpha measures internal consistency reliability that assumes the same thresholds. Results between 0.70 and 0.95 represent “satisfactory to good” reliability levels.

Next, the convergent validity was calculated, which is the extent to which a construct converges in its indicators by explaining the items’ variance. Convergent validity is assessed by the average variance extracted (AVE) across all items associated with a particular construct and is also referred to as communality. An acceptable threshold for the AVE is 0.50 or higher. This level or higher indicates that, on average, the construct explains (more than) 50% of the variance of its items.

The last step in reflective measurement is to assess discriminant validity. This analysis reveals to which extent a construct is empirically distinct from other constructs both in terms of how much it correlates with other constructs and how distinctly the indicators represent only this single construct. Discriminant validity assessment in PLS-SEM involves analyzing Henseler et al.’s (2015) [46] heterotrait–monotrait ratio (HTMT) of correlations. The suggested threshold is a value of 0.90, when the path model included constructs that are conceptually very similar. Our model presents this concept. The heterotrait–monotrait ratio of correlations is a new criterion to assess the discriminant validity in variance-based structural equation modeling, which is superior compared with the Fornell–Larcker criterion and (partial) cross-loadings.

### 2.3. Model

**Figure 1:**
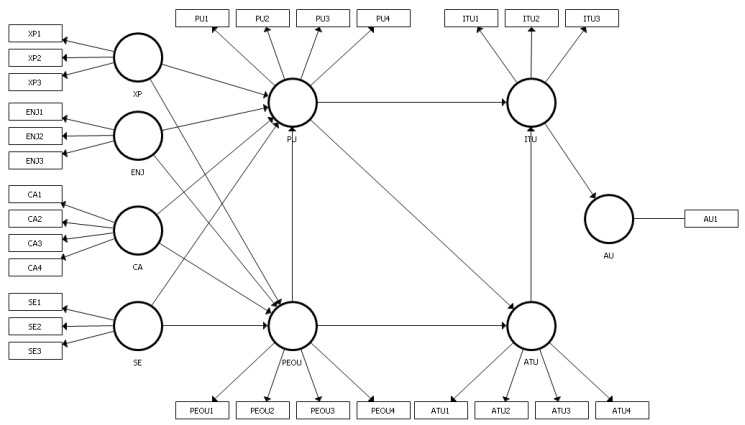
Distance learning model. XP: Experience, ENJ: Enjoyment, CA: Computer Anxiety, SE: Self-efficacy, PU: Perceived Usefulness, PEOU: Perceived Ease of Use, ITU: Intention to Use, ATU: Attitude Towards Using, AU: Actual Use.

Figure 1 shows the SmartPLS 3 (SmartPLS GmbH, Bönningstedt, Germany) model that we adopted for the hypotheses connected with distant learning [47]. The model is adapted from Abudallah and Ward (2016) [38] and was originally presented as a General Extended Technology Acceptance Model for E-Learning (GETAMEL). We use this model as the basis for further research, with some modifications. Namely, we have resigned from subjective norms (SN) construct due to the mandatory character of distance courses during COVID-19 pandemic. This model combines the Technology Acceptance Model (TAM) with external factors. In this study, the following external factors are used: Experience (XP), Enjoyment (ENJ), Computer Anxiety (CA), and Self-Efficacy (SE).

In terms of construct measurement, all the constructs have reflectively specified measurement models with three to four items. The Actual Use draws on a single-item measure. Table 1 provides an overview of all items’ survey questions.

Using five items with reversed intention allowed us to have control in the survey for consistency of answers.

## 3. Results

The data for research were collected through a survey in Google Forms. Before the final publication, the questionnaire was trialed on a small sample of respondents who were students familiar with the PLS-SEM method. After testing, the final set of questions was adjusted, and the questionnaire was sent to all students from the University of Economics in Katowice. The survey was published on 13 May 2020 and it remained opened until 4 June 2020. However, the majority of responses came during the first three days. All 7130 active students of UEK were invited to participate in the study by sending an individual email invitation. The survey allowed collecting 1692 responses or 23.7% of the student population at UEK. The structure of respondents is presented in Table 2. Bachelor’s level is the first study cycle, and Master is the second. The students of full-time studies attend university from Monday to Friday; part-time students have courses on weekends.

Next, the data were screened. There were no missing values since it was guaranteed by the structure of the survey. We excluded 27 answers because respondents marked the same answer for each question and the calculated variance was 0. Finally, 1665 data rows were used for calculation. This sample size is sufficient for the PLS path model estimation.

**Figure 2:**
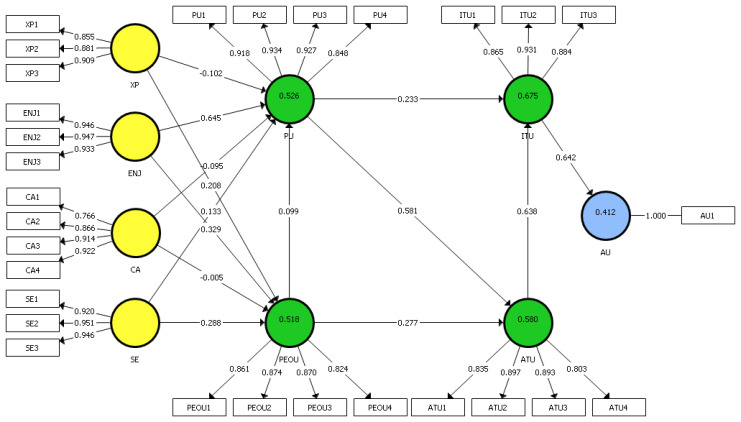
Distance learning model and PLS-SEM results. XP: Experience, ENJ: Enjoyment, CA: Computer Anxiety, SE: Self-efficacy, PU: Perceived Usefulness, PEOU: Perceived Ease of Use, ITU: Intention to Use, ATU: Attitude Towards Using, AU: Actual Use.

The model estimation applied in the research uses the basic PLS-SEM algorithm, the centroid weighting scheme, the maximum of 300 iterations, the stop criterion of 1 × 10^−7^, and equal indicator weights for the initialization. Figure 2 shows the PLS-SEM results. The numbers on the path relationships represent the standardized regression coefficients, while the numbers displayed in the circles of the constructs represent the *R*^2^ values.

Table 3 shows the results and evaluation of criteria outcomes. All reflective measurement models were found to meet the relevant assessment criteria. More specifically, all the outer loadings are above 0.70, indicating that all indicators exhibit a sufficient level of reliability (i.e., >0.50). Further, all AVE values were above 0.50, providing support for the measures’ convergent validity. Composite reliability had values of 0.865 and higher, which is clearly above the expected minimum level of 0.70. Moreover, the Cronbach’s alpha values ranged between 0.867 and 0.937, which is acceptable. Finally, all ρA values met the 0.70 threshold. These results suggest that the construct measures of XP, ENJ, CA, SE, PEOU, PU, ATU, and ITU exhibit sufficient levels of internal consistency reliability.

Finally, the discriminant validity was evaluated using the HTMT criterion. All results were below the threshold of 0.90, except ITU to ATU, which was 0.905 (Table 4), but this is still an acceptable result for this model. Next, the bootstrapping procedure with 5000 samples was run, and the “no sign” changes option was used together with the BCa bootstrap confidence intervals and two-tailed testing at the 0.05 significance level. The results show that none of the HTMT confidence intervals include value 1, suggesting that all the HTMT values are significantly different from 1. We thus conclude that discriminant validity has been established.

The effects for path coefficients, which we have obtained, are presented in Table 5. Thirteen hypotheses were found to be significant at 5% error level, whereas one effect was not significant and hypothesis 12 was not supported.

Multigroup analysis with PLS-SEM, which tests a single structural relationship at a time, is an effective way to evaluate moderation across multiple relationships versus standard moderation [49]. We selected three variables of interest: gender, study type, and study level. The groups are large enough for statistical power.

## 4. Discussion

The initial assessment of the core TAM variables (Table 5) shows that Attitude Towards Using (ATU) has the strongest effect (0.638) on Intention to Use (ITU) (H5), followed by Perceived Usefulness (PU) (0.233) (H2). These two constructs explain 67.5% (i.e., the *R*^2^ value) of the variance of the ITU construct. The PU has the strongest effect (0.581) on ATU (H1) followed by Perceived Ease of Use (PEOU) (0.277) (H4), and these two constructs explain 58% of the variance of the construct ATT. The ITU has a strong effect of 0.642 on Actual Use (AU) (H6) which explains 41% of the variance. Bootstrapping results substantiate that the effects of PU and ATU on the ITU are significant and the effects of PEOU and PU on the ATU are also significant at the 5% probability of error level. The authors also find that the model explains 51.8% of PEUO’s variance and 52.6% of PU’s variance, which is relatively high.

When analyzing the key predictors of PU, which has a substantial *R*^2^ value of 0.526, the authors find that Enjoyment (ENJ) has the strongest significant effect (0.645) (H9), followed by Self-Efficacy (SE) (0.133) (H13) and two negative effects of Experience (XP) (−0.102) (H7) and Computer Anxiety (CA) (−0.095) (H11). The XP and CA have a negative effect because of the presence of a very strong predictor, which in this model, turned out to be ENJ. All effects are significant at the 5% level. PEOU also has a substantial *R*^2^ value of 0.518. Analysis of this construct’s predictors shows that ENJ (0.329) (H10), SE (0.288) (H14), and XP (0.208) (H8) have the strongest significant effect. On the contrary, the effect of CA (−0.005) (H12) on PEOU is not significant at the 5% level. Table 5 also shows the *f*^2^ effect sizes. Relatively high *f*^2^ effect sizes occur for the relationships ITU on AU (0.777), ATU on ITU (0.596), PU on ATU (0.592), and ENJ on PU (0.138). These relationships also have particularly strong path coefficients of 0.58 and higher.

Using the results from the multigroup analysis, we observed no significant difference between the opinions of female and male (Table 6) respondents. For the study level, there is a significant difference for the two path coefficients. Master students have a stronger effect of ENJ on PEOU and of PEOU on ATU than bachelor students (Table 7). For the study type, there is a significant difference for one path coefficient. Part-time students have a stronger effect of ITU to AU then full-time students (Table 8).

From our point of view, as of 13 July, the University of Economics in Katowice, represented by its authorities, administrative staff, academic teachers and, of course, students, have faced the pandemic situation with dignity, coming through a complex process of adaptation to realities of distance learning and ensuring the same high quality of education even remotely. On par with universities around the globe, UEK has been keeping higher education afloat during this tough time [50,51]. However, for now, there are no official data on the feedback of students and teachers on their successes and failures when learning/teaching distantly. UEK authorities launched a survey process for students at the end of the semester (which ended on 28 June with the exam session) yet, presently, there is no information on when and how the results will be distributed.

One of the issues connected with the shift to distance education is the teaching process itself, transferred to online communication platforms [52]. We conducted a preliminary investigation of UEK students (*n* = 1692) to discover how they perceive this forced shift to distance education.

### 4.1. Practical Implication

It can be concluded that students have a low to medium feeling that within around two months (since 12 March 2020), distance learning enhanced their effectiveness, improving course performance and productivity. Their self-efficacy with distance learning, expressed as confidence, is medium to medium-high. Nevertheless, students consider distance learning tools and software to be very intuitive; understanding the principles of this educational form does not cause problems for them. They are generally comfortable with using computers and the internet; they consider distance learning to be a good idea and plan to use it often during the semester. This might be also conditioned by the fact that students have experience of taking additional online courses on specific platforms (e.g., Coursera (Stanford University, Mountain View, CA, USA), Udemy (Udemy Inc., San Francisco, CA, USA), edX (Harvard University, Massachusetts University of Technology, Cambridge, USA), etc.).

However, with the use of computers, there comes the problem of the software to be installed on them. Some courses at UEK (as, of course, at other HEIs) presuppose usage of specific software which can only be used at a university that possesses a license. Additionally, it turned out that some students use only Google Documents (Google, Mountain View, CA, USA) and Google Sheets (Google, Mountain View, CA, USA) or Microsoft OneDrive (Microsoft Corporation, Redmond, WA, USA). Such problems with software have made university teachers adjust to such a reality and amend their course plans to make them suitable for all students. It is necessary to add that changes in the traditional methodology and strategy of teaching turned out to be confusing for some teachers, which might have caused a slight reduction in the quality of teaching compared to the traditional mode of studies.

Despite the abovementioned opinions about the general comfort and usefulness of online education, the students indicated they would definitely like to go back to traditional education—to the University campus where they can communicate with their friends and to the classrooms where they can discuss various issues with their teachers, giving and getting feedback from a person and not from a computer screen, to the library where they can study and read paper books. They felt confused by the idea of having to pass course exams via online platforms and, moreover, by having to pass diploma exams and present their work to the commission online. There is no doubt that these procedures are always stressful for students, and now their emotions will be only heightened by the anxiety of being one on one with the computer screen.

At the end of the summer semester 2019/20, all courses taught in distance form at the University of Economics in Katowice were evaluated by the students. It is expected that this evaluation will provide reliable and helpful feedback on the subject of online classes, and this, in turn, will contribute to improving the quality of education at UEK—both online and traditional.

### 4.2. Contribution

The research described in this paper was conducted in the period of the coronavirus pandemic, which has covered the whole world and has not left a single country uninvolved. All educational institutions were caught by surprise and had to throw all their efforts toward adjusting to the new reality as quickly as possible. The survey “caught” the students of a university in the middle of the period of distance learning, to which they all had to switch. Such timing allowed getting the most state-of-the-art feedback from students as for the methods and tools used in the process and exploring their emotions while they were still experiencing them. The authors consider picturing such state-of-the-art students’ attitude to be one of the contributions of this research. To the authors’ best knowledge, this is the first study testing the shift to distance learning using the adapted GETAMEL model and PLS-SEM method.

Since it is very likely that in the following semester (winter 2020/2021), universities worldwide are going to work in a distance form (or, at least, partially), the authors consider results of the research to be useful for universities policymakers. It is important that introduction of any new distance learning tool, as well any learning technique or rule, is done gradually, with proper preparation. Attitude Towards Using, which is one of the strongest acceptance variables, is an emotional component, influenced among others by how a technology is presented to students—i.e., the first impression is very important. The authors also hope that responses of students of the University of Economics will help University’s authorities adjust (as much as possible) their policy for the new semester to students’ attitudes.

### 4.3. Limitations

The article contains a brief observation of the situation which higher education institutions in Poland were forced to face because of the pandemic situation since March 2020. Based on the example of a single university in the country, the article cannot be positioned as giving a full picture of what higher education is going through. However, the authors consider that in the current situation, sharing experience is valuable, and each particular university contributes very much to the global struggle with the new reality. The authors consider it reasonable and important to conduct more thorough research on the experience of educational institutions in the country, examining more cases. The authors also express their deep conviction that after the pandemic, when the life and work get back to normal (with no restrictions and threats to health), the overall level of education in Poland, as well as all over the world, will be even higher, improved by the new valuable experience.

## 5. Conclusions

The research, conducted in this paper, is based on a survey conducted among full- and part-time students of the University of Economics in Katowice (Poland), in May–June 2020, when all universities had to shift to distance learning because of the coronavirus outbreak. The survey has allowed for analyzing students’ acceptance of distance learning and, particularly, of the IT tools applied by the University in the process. It was found out that students have a medium feeling that distance learning has been enhancing their effectiveness and productivity; their self-efficacy with distance learning is also medium; students consider distance learning IT tools to be very intuitive, and they are generally comfortable with using computers and the internet; they plan to use distance learning often during the semester. However, despite the positive opinions about distance education, the students would like to go back to traditional education. This research is a valuable contribution for policy-making in case the COVID-19 situation forces HEIs to continue working online. Nevertheless, this work has limitations since only one university in a single country was observed. Realization of comparative research would be reasonable to get a wider picture of the impact of pandemic on higher education.

## Figures and Tables

**Table 1:** Items used in the survey.

Perceived Usefulness (PU) [35,48]	
PU1	Using distance learning would enhance my effectiveness in studying.
PU2	Using distance learning would improve my course performance.
PU3	Using distance learning would improve my productivity in courses.
PU4	I find distance learning useful for my studies.
**Perceived Ease of Use (PEOU) [35,48]**	
PEOU1	I find distance learning easy to use.
PEOU2	Mastering distance learning would be easy for me.
PEOU3	My interaction with distance learning is clear and understandable.
PEOU4	It would be easy for me to find the required information using distance learning.
**Attitude Towards Using (ATU) [35,48]**	
ATU1	I dislike the idea of using distance learning. (R)
ATU2	I have a generally favorable attitude towards using distance learning.
ATU3	I believe it is (would be) a good idea to use distance learning for my study process.
ATU4	Using distance learning is a foolish idea. (R)
**Intention to Use (ITU) [35,48]**	
ITU1	I intend to use distance learning during the semester.
ITU2	I will return to distance learning often.
ITU3	I intend to use distance learning frequently for my study process.
**Actual Use (AU) [48]**	
AU	I use distance learning frequently.
**Experience (XP) [32] and own**	
XP1	I enjoy using computers.
XP2	I am comfortable using the internet.
XP3	I am comfortable saving and locating files.
XP4	I am comfortable with using software for distance learning.
**Enjoyment (ENJ) [32]**	
ENJ1	I find distance learning process enjoyable.
ENJ2	The actual process of using distance learning is pleasant.
ENJ3	I have fun using distance learning.
**Computer Anxiety (CA) [32]**	
CA1	Computers do not scare me at all.
CA2	Computers make me feel uncomfortable. (R)
CA3	Working with computer makes me nervous. (R)
CA4	Computers make me feel uneasy. (R)
**Self-Efficacy (SE) [32]**	
SE1	I am confident of using distance learning even if there is no one around to show me how to do it.
SE2	I am confident of using distance learning even if I have never used such a system before.
SE3	I am confident of using distance learning even if I have only the software manuals for reference.

Note: R = reversed item intention.

**Table 2:** Structure of the respondents.

Gender	Count	Percentage
Female	1220	72.1%
Male	472	27.9%
**Study level**		
Bachelor	1283	75.8%
Master	409	24.2%
**Study type**		
Full-time	1269	75%
Part-time	423	25%

**Table 3:** PLS-SEM assessment results of measurement models.

Latent Variable	Indicators	Convergent Validity			Internal Consistency Reliability		
		Loadings	Indicator Reliability	AVE	Composite Reliability ρc	Reliability ρA (rho_A)	Cronbach’s Alpha
		>0.70	>0.50	>0.50	>0.70	>0.70	0.70–0.95
ATU	ATU1	0.835	0.697	0.736	0.917	0.893	0.880
	ATU2	0.897	0.805				
	ATU3	0.893	0.797				
	ATU4	0.803	0.645				
CA	CA1	0.766	0.587	0.755	0.925	0.894	0.890
	CA2	0.866	0.750				
	CA3	0.914	0.835				
	CA4	0.922	0.850				
ENJ	ENJ1	0.946	0.895	0.888	0.960	0.938	0.937
	ENJ2	0.947	0.897				
	ENJ3	0.933	0.870				
ITU	ITU1	0.865	0.748	0.799	0.923	0.880	0.874
	ITU2	0.931	0.867				
	ITU3	0.884	0.781				
PEOU	PEOU1	0.861	0.741	0.736	0.918	0.881	0.880
	PEOU2	0.874	0.764				
	PEOU3	0.870	0.757				
	PEOU4	0.824	0.679				
PU	PU1	0.918	0.843	0.824	0.949	0.929	0.928
	PU2	0.934	0.872				
	PU3	0.927	0.859				
	PU4	0.848	0.719				
SE	SE1	0.920	0.846	0.882	0.957	0.935	0.933
	SE2	0.951	0.904				
	SE3	0.946	0.895				
XP	XP1	0.855	0.731	0.778	0.913	0.995	0.867
	XP2	0.881	0.776				
	XP3	0.909	0.826				

XP: Experience, ENJ: Enjoyment, CA: Computer Anxiety, SE: Self-efficacy, PU: Perceived Usefulness, PEOU: Perceived Ease of Use, ITU: Intention to Use, ATU: Attitude Towards Using, AU: Actual Use.

**Table 4:** Heterotrait–monotrait ratio (HTMT) values.

Variable	ATU	AU	CA	ENJ	ITU	PEOU	PU	SE	XP
ATU									
AU	0.564								
CA	0.313	0.192							
ENJ	0.852	0.556	0.362						
ITU	0.905	0.687	0.273	0.851					
PEOU	0.649	0.432	0.403	0.721	0.622				
PU	0.788	0.530	0.161	0.755	0.762	0.564			
SE	0.671	0.471	0.464	0.738	0.644	0.702	0.579		
XP	0.523	0.361	0.728	0.644	0.543	0.617	0.376	0.616	

**Table 5:** Path coefficient of the structural model and significance testing results.

Hypothesis	Path	Path Coefficient	95% BCa Confidence Interval	*f*^2^ Effect Size	Significant (*p* < 0.05)?
H1	PU → ATU	0.581	[0.545–0.616]	0.592	Yes
H2	PU → ITU	0.233	[0.190–0.276]	0.079	Yes
H3	PEOU → PU	0.099	[0.048–0.149]	0.010	Yes
H4	PEOU → ATU	0.277	[0.240–0.317]	0.135	Yes
H5	ATU → ITU	0.638	[0.597–0.676]	0.596	Yes
H6	ITU → AU	0.642	[0.609–0.674]	0.700	Yes
H7	XP → PU	−0.102	[−0.158–−0.048]	0.008	Yes
H8	XP → PEOU	0.208	[0.149–0.266]	0.036	Yes
H9	ENJ → PU	0.645	[0.590–0.691]	0.352	Yes
H10	ENJ → PEOU	0.329	[0.276–0.380]	0.099	Yes
H11	CA → PU	−0.095	[−0.138–−0.051]	0.011	Yes
H12	CA -> PEOU	−0.005	[−0.053–0.043]	0.000	No
H13	SE → PU	0.133	[0.081–0.186]	0.016	Yes
H14	SE → PEOU	0.288	[0.235–0.343]	0.080	Yes

**Table 6:** Multigroup analysis results for gender.

Path	Female		Male		Difference	
	Path Coefficients	Significant (*p* < 0.05)	Path Coefficients	Significant (*p* < 0.05)	Path Coefficients (Female–Male)	Significant?
ATU → ITU	0.657	Yes	0.597	Yes	0.060	No
CA → PEOU	0.003	No	−0.007	No	0.011	No
CA → PU	−0.099	Yes	−0.082	Yes	−0.017	No
ENJ → PEOU	0.290	Yes	0.363	Yes	−0.072	No
ENJ → PU	0.654	Yes	0.612	Yes	0.042	No
ITU → AU	0.630	Yes	0.664	Yes	−0.034	No
PEOU → ATU	0.294	Yes	0.242	Yes	0.052	No
PEOU → PU	0.090	Yes	0.118	Yes	−0.028	No
PU → ATU	0.578	Yes	0.583	Yes	−0.006	No
PU → ITU	0.214	Yes	0.264	Yes	−0.050	No
SE → PEOU	0.296	Yes	0.279	Yes	0.018	No
SE → PU	0.147	Yes	0.107	Yes	0.039	No
XP → PEOU	0.237	Yes	0.176	Yes	0.060	No
XP → PU	−0.117	Yes	−0.054	No	−0.063	No

**Table 7:** Multigroup analysis results for study level.

Path	Bachelor		Master		Difference	
	Path Coefficients	Significant (*p* < 0.05)	Path Coefficients	Significant (*p* < 0.05)	Path Coefficients (Bachelor–Master)	Significant?
ATU → ITU	0.641	Yes	0.619	Yes	0.022	No
CA → PEOU	0.007	No	−0.048	No	0.055	No
CA → PU	−0.092	Yes	−0.102	Yes	0.010	No
ENJ → PEOU	0.287	Yes	0.470	Yes	−0.183	Yes
ENJ → PU	0.620	Yes	0.724	Yes	−0.104	No
ITU → AU	0.630	Yes	0.677	Yes	−0.047	No
PEOU → ATU	0.258	Yes	0.348	Yes	−0.090	Yes
PEOU →PU	0.111	Yes	0.039	No	0.073	No
PU → ATU	0.579	Yes	0.570	Yes	0.009	No
PU → ITU	0.216	Yes	0.287	Yes	−0.071	No
SE → PEOU	0.301	Yes	0.238	Yes	0.063	No
SE → PU	0.153	Yes	0.070	No	0.083	No
XP → PEOU	0.221	Yes	0.156	Yes	0.065	No
XP → PU	−0.113	Yes	−0.070	No	−0.043	No

**Table 8:** Multigroup analysis results for study type.

Path	Full-Time		Part-Time		Difference	
	Path Coefficients	Significant (*p* < 0.05)	Path Coefficients	Significant (*p* < 0.05)	Path Coefficients (Full-Time–Part-time)	Significant?
ATU → ITU	0.635	Yes	0.651	Yes	−0.016	No
CA → PEOU	−0.011	No	0.031	No	−0.042	No
CA → PU	−0.106	Yes	−0.051	No	−0.055	No
ENJ → PEOU	0.326	Yes	0.339	Yes	−0.013	No
ENJ → PU	0.633	Yes	0.692	Yes	−0.058	No
ITU → AU	0.615	Yes	0.730	Yes	−0.115	Yes
PEOU → ATU	0.292	Yes	0.233	Yes	0.059	No
PEOU → PU	0.081	Yes	0.123	Yes	−0.042	No
PU → ATU	0.570	Yes	0.617	Yes	−0.047	No
PU → ITU	0.235	Yes	0.233	Yes	0.002	No
SE → PEOU	0.276	Yes	0.320	Yes	−0.044	No
SE → PU	0.166	Yes	0.049	No	0.117	No
XP → PEOU	0.239	Yes	0.128	Yes	0.111	No
XP → PU	−0.101	Yes	−0.099	No	−0.002	No
